# Novel Asynchronous Emergency Medicine Sub-Internship Curriculum Utilizing Free Open Access Medical Education (FOAM)

**DOI:** 10.21980/J8.52135

**Published:** 2025-10-31

**Authors:** Sophia Murphy, Courtney Kim, Tomas Diaz, Jimmy Truong, Emmagene Worley

**Affiliations:** * Columbia University Vagelos College of Physicians and Surgeons, Department of Emergency Medicine, New York, NY

## Abstract

**Audience and Type of Curriculum:**

This emergency medicine asynchronous curriculum is designed for emergency medicine sub-interns.

**Length of Curriculum:**

The curriculum runs monthly over a four-week sub-internship rotation.

**Introduction:**

Emergency medicine sub-interns are at an intermediate stage of training and require exposure to material that agrees with their training level. Asynchronous learning is an effective way to supplement sub-interns' learning, and free open-access medical education (FOAM) provides valuable content for asynchronous curricula.

**Educational Goals:**

The global purpose of the curriculum is to supplement sub-interns' learning with high-yield emergency medicine topics while introducing them to various FOAM resources.

**Educational Methods:**

A gap-analysis was conducted to suggest content most appropriate for the asynchronous curriculum, and the curriculum was designed with adult learning theories in mind. The educational strategies used in the curriculum include articles, videos, podcasts, diagrams, cases, and practice questions from various high-quality FOAM resources. The content is broken up into four core modules (trauma, eye complaints, shock, obstetric and gynecologic (OBGYN) complaints) and two bonus modules (orthopedic complaints, ultrasound basics), and the modules are available on the sub-internship website for students to access throughout their rotation.

**Research Methods:**

The educational content was evaluated by the learners using pre-rotation, post-module, and post-rotation surveys.

**Results:**

Survey results show that at least 95% of students agreed with meeting each of the modules’ learning objectives. The curriculum also significantly increased learners’ confidence in evaluating specific chief complaints and ultrasounds. Students reported an increased likelihood of changing their clinical practice, an increased awareness of the role of social determinants of health, and an increased preparedness for residency after engaging with the curriculum.

**Discussion:**

Asynchronous curricula using thoughtfully-selected FOAM content and resources can effectively supplement synchronous learning methods in emergency medicine sub-internships. The curriculum is easy to implement and receives high satisfaction from students.

**Topics:**

Sub-Internship, asynchronous curriculum, free-open access medical education, trauma, eye complaints, shock, OBGYN complaints, ultrasound, social determinants of health.

## USER GUIDE

**Table:** 

List of Resources:	
Abstract	1
User Guide	3
Didactics and Hands on Curriculum Chart	12
Asynchronous Curriculum	15
Surveys	25

**Learner Audience:**

Medical students

**Length of Curriculum:**

The curriculum is run monthly over a four-week long sub-internship rotation.

**Topics:**

Sub-Internship, asynchronous curriculum, free-open access medical education, trauma, eye complaints, shock, OBGYN complaints, ultrasound, social determinants of health.

**Objectives:**

By the end of this curriculum learners will be able to:

General Curriculum Learning Objectives

1. Prepare students to evaluate patients with presentations related to trauma, eye complaints, shock, and OBGYN complaints
2. Expose students to various emergency medicine resources
3. Expand students’ awareness of the relationship between social determinants of health and presentations to the emergency department

Trauma Module Learning Objectives

1. Describe the primary and secondary survey
2. Identify common abnormal findings requiring timely interventions

Eye Complaints Module Learning Objectives

1. Describe the major categories of eye complaints, common diagnoses, and can’t miss diagnoses
2. Gather an adequate history for eye complaints
3. Become familiar with the slit lamp exam
4. Describe the functions of bedside ocular ultrasound

Shock Module Learning Objectives

1. Describe the types of shock and common causes of each
2. Determine appropriate workup for undifferentiated shock
3. List the components of a rapid ultrasound for shock and hypotension (RUSH) exam
4. Decide on management for each type of shock

OBGYN Module Learning Objectives

1. Describe the clinical presentation of a ruptured ectopic
2. Develop an approach for evaluating vaginal bleeding
3. Describe the clinical presentation and initial management of pre-eclampsia

### Brief introduction

It is important that medical students learn the basics of recognizing and managing medical emergencies. In 1995, the Macy Foundation Report for Emergency Medicine voiced this sentiment and encouraged organizations including the Liaison Committee on Medical Education (LCME) and National Board of Medical Examiners (NBME) to ensure this.^1^ Since this recommendation, several groups have outlined standardized emergency medicine curricula for medical students.^2^–^7^ These curricula detail the knowledge and skills that all medical students should acquire over their four years of medical school or during their required emergency medicine clerkship. Some of the curricula also detail the format in which the content should be taught. There is, however, a paucity of literature on the development of emergency medicine curricula for sub-internships. 8–12 Given the breadth of EM content, the variability of clinical exposures at each sub-I program, and the constraints of a short 4-week rotation, there is no consensus on the most effective EM sub-internship curriculum. In fact, in 2010 when the Clerkship Directors in Emergency Medicine (CDEM) modified its syllabi to standardize the EM learning experience of medical students nationwide, they intentionally left flexibility for institutions to teach to their strengths. As sub-interns are the future of the emergency medicine workforce, it is vital to continue curriculum development to ensure they receive appropriate training to meet the needs of our patients.

### Problem identification, general and targeted needs assessment

Emergency medicine sub-interns are at an intermediate stage of training and require exposure to topics that meet their training level using contemporary methods that agree with an academic institution’s schedule. Emergency medicine sub-interns typically learn in a variety of settings including clinical shifts, didactic sessions, and conferences. There is an opportunity to augment sub-interns' growth by providing additional learning opportunities. Many studies suggest the value of active synchronous learning in medical education.^13^–^16^ More recently, there has been a movement towards supplementing synchronous learning with asynchronous learning.^13^,^17^–^22^ Several groups have shown that asynchronous medical school curricula are effective and result in similar student performance and satisfaction when compared to synchronous curricula.^23^–^25^. The flexibility of asynchronous learning is especially appealing given scheduling constraints of many academic institutions. Many options for asynchronous learning material exist. There has been a recent explosion in FOAM, especially in emergency medicine and critical care.^26^–^28^ The growth in these fields is especially important due to the breadth of knowledge needed to care for these patients and the generalizability of the content to other fields. The implications of FOAM are significant in its ability to increase access to high quality medical education resources and to increase collaborations between institutions.^29^,^30^ FOAM also allows for learners to indulge in self-directed learning, keeps learners engaged through interactive components, and provides the opportunity for students to individualize their education based on personal interests. A previous group created an asynchronous curriculum for emergency medicine learners using existing FOAM resources; however, from our knowledge, no such curriculum has been created for emergency medicine sub-interns.^22^

**Figure 1:**
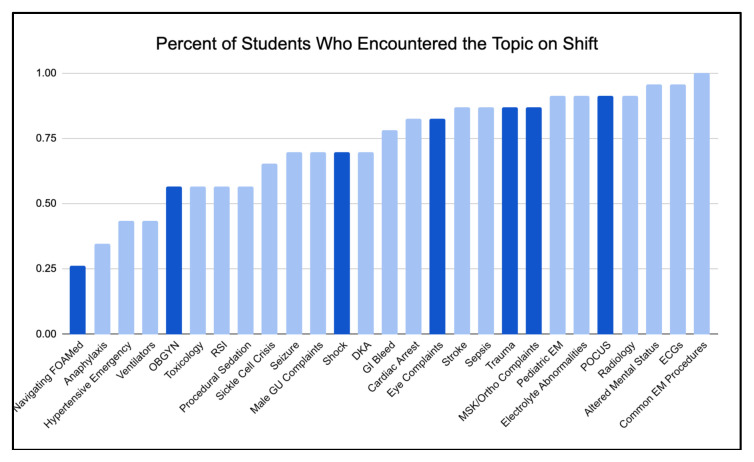
Percent of Students Who Encountered the Topic on Shift

**Figure 2:**
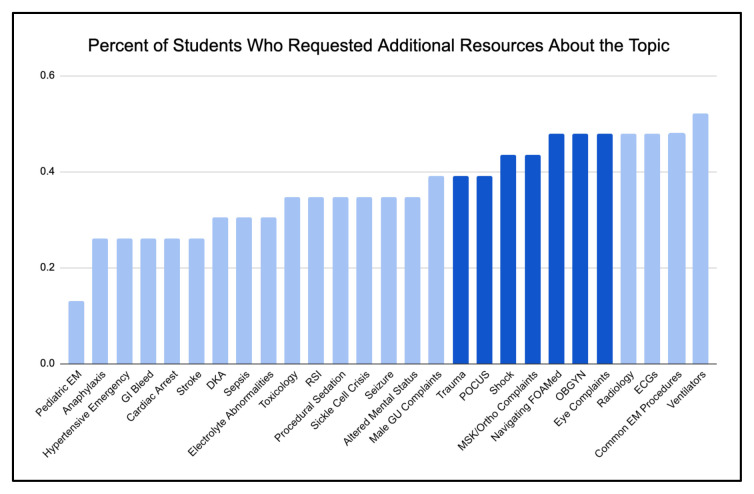
Percent of Students Who Requested Additional Resources About the Topic

We used the ADDIE model for curriculum development to Analyze, Design, Develop, Implement, and Evaluate our asynchronous curriculum over multiple years. The previous sub-intern curriculum topics were chosen to target chief complaints often triaged to areas of our institution’s emergency department where sub-interns are not assigned shifts; however, it was unclear whether these were the highest yield topics for our learners. Along with a literature review, we performed a gap-analysis on our previous sub-internship curriculum to identify both content and design gaps. From this, we determined the topics, length, and format that would be best to include in our new supplemental asynchronous curriculum. There was a total of twenty-three stakeholders surveyed for the gap-analysis which included faculty, residents, and sub-interns. Our survey collected data on the perceived usefulness of various formats of didactic curricula that sub-interns experienced at both their home and away rotations. We compiled a list of common EM topics identified in the literature and for each topic, surveyed whether it was encountered clinically and whether students would find additional resources useful (Figures 1 & 2). For common EM resources and FOAM, we surveyed whether students were familiar with each resource and whether they found that resource useful. Lastly, we allowed students to input free responses regarding other resources they would recommend, their ideal format of didactic curriculum, and any aspect of the curriculum that they did not find helpful. The survey was distributed via Google Forms and is included in Supplementary A.

Many topics were suggested by learners, but those chosen were determined to be high yield for our learners based on quality of exposure and requested resources. The four core topics chosen from the gap-analysis were trauma, eye complaints, shock, and OBGYN complaints, along with two bonus topics (orthopedic complaints, ultrasound basics) and two longitudinal topics (ultrasound applications and social determinants of health). Trauma was selected because our hospitals have limited level 1 trauma care, OBGYN and eye complaints were selected because these chief complaints tend to go to fast-track areas where students have fewer shifts, shock was selected because students tend to be on the periphery of managing patients in critical conditions, orthopedic complaints were selected because they are broad and frequently seen in the emergency department, ultrasound was selected because it is a rapidly growing tool within emergency medicine, and social determinants of health was selected because in our urban environment, social context is a vital part of patient care and disposition. Nuances of our institution point out the necessity of programs to design a curriculum befitting their institutions’ unique structures, gaps, and strengths.

There were a few topics for which students requested additional resources that were not chosen as core topics because they were either covered in other parts of the overall curriculum (ECGs, radiology, EM procedures) or were determined to not be high-yield for learners’ current level (ventilators). In the surveys, both residents and sub-interns also noted their lack of comfort with navigating FOAM resources. With the topics in mind, a variety of high-quality FOAM resources were selected to form the content of the curriculum. From these resources, different modalities, including articles, videos, podcasts, diagrams, cases, and practice questions were utilized to accomplish learning objectives.

In our design, we considered theories of adult learning to maximize curriculum effectiveness: we provided opportunities for learners to identify knowledge gaps, used learning objectives to guide relevance of information, included opportunities for feedback through interactive modules and assessments, and allowed for reflection through post-module surveys.^31^ When creating learning objectives, we aimed to set SMART (specific, measurable, achievable, relevant, timed) goals using the highest level of Bloom’s Taxonomy.^32^ To assess whether learning objectives were met, we aimed to utilize the highest feasible level of Miller’s pyramid.^31^ Due to limitations of facilities and faculty time, this was done through interactive cases and question banks, which had the benefit of providing learners independence and the opportunity to practice self-assessment..

### Goals of the curriculum

The global purpose of the curriculum is to supplement sub-interns' learning with high-yield emergency medicine topics while introducing them to various FOAM resources.

### Objectives of the curriculum

By the end of this curriculum learners will be able to:

General Curriculum Learning Objectives

1. Prepare students to evaluate patients with presentations related to trauma, eye complaints, shock, and OBGYN complaints
2. Expose students to various emergency medicine resources
3. Expand students’ awareness of the relationship between social determinants of health and presentations to the emergency department

Trauma Module Learning Objectives

1. Describe the primary and secondary survey
2. Identify common abnormal findings requiring timely interventions

Eye Complaints Module Learning Objectives

1. Describe the major categories of eye complaints, common diagnoses, and can’t miss diagnoses
2. Gather an adequate history for eye complaints
3. Become familiar with the slit lamp exam
4. Describe the functions of bedside ocular ultrasound

Shock Module Learning Objectives

1. Describe the types of shock and common causes of each
2. Determine appropriate workup for undifferentiated shock
3. List the components of a rapid ultrasound for shock and hypotension (RUSH) exam
4. Decide on management for each type of shock

OBGYN Module Learning Objectives

1. Describe the clinical presentation of a ruptured ectopic
2. Develop an approach for evaluating vaginal bleeding
3. Describe the clinical presentation and initial management of pre-eclampsia

### Educational Strategies

See curriculum chart

### Results and tips for successful implementation

#### Implementation and method of evaluation

The asynchronous curriculum is available on the sub-internship's website (Addendum). While it was designed for students to complete one of the four required modules every week, students have the flexibility to complete the modules at their own pace. The curriculum’s effectiveness was assessed using pre-rotation, post-rotation, and post-module surveys which gathered demographic information and addressed the curriculum’s learning objectives (Supplementary A). The surveys were also available on the sub-internship's website and were sent out to students at the beginning and end of the four-week rotation. Pre-post survey data was considered paired, and mean difference scores were evaluated using the Student’s t-test (one tailed, significance *p<0.01). The study was deemed institutional review board (IRB) exempt at New York-Presbyterian Brooklyn Methodist.

#### Survey completion and participant demographics

**Figure 3:**
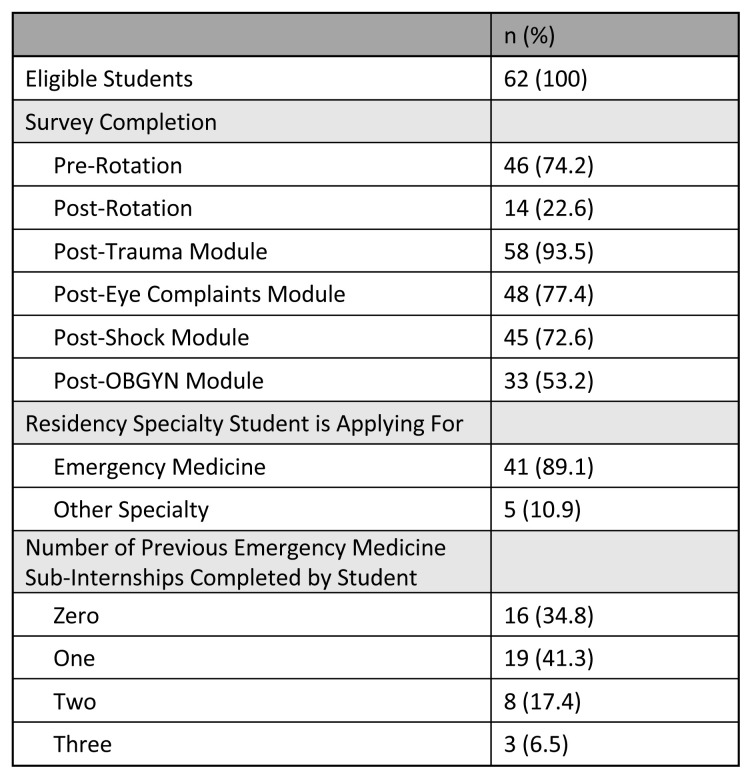
Survey Completion and Participant Demographics

Seven cohorts of students from April to October 2023, totaling 62 students, were eligible to participate in this study. Student participation varied by survey, and the majority of students engaging with the surveys were planning to apply into emergency medicine residency (Figure 3). The largest portion of students were participating in their second sub-internship, but this response ranged from zero to three previous emergency medicine sub-internships completed. (Figure 3).

#### Effectiveness of modules in addressing learning objectives and changing clinical practice

**Figure 4:**
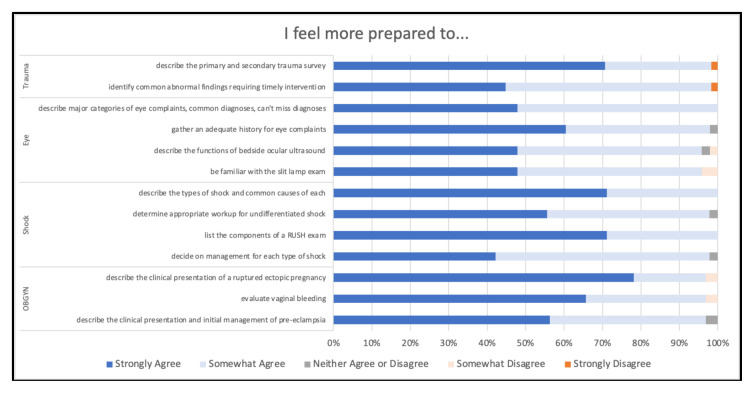
Preparedness to Address Learning Objectives

In post-module surveys, at least 95% of students agreed to feeling more prepared to address each of the modules’ learning objectives (Figure 4). Therefore, very few students (<5%) disagreed or neither agreed nor disagreed with feeling more prepared to address the modules’ learning objectives, and there was no apparent pattern in learning objectives that received these responses.

**Figure 5:**
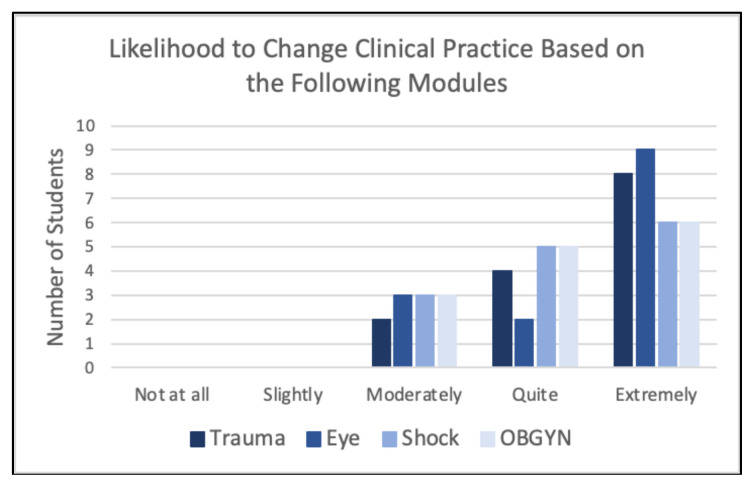
Likelihood to Change Clinical Practice

In post-rotation surveys, when assessing whether the modules had influence on participants’ clinical practice, the majority of participants (>50%) stated they were “extremely likely” or “quite likely” to change their clinical practice based on the four core modules (Figure 5). No student responded that they were “not at all” likely to change their clinical practice based on the four main modules.

#### Effectiveness of curriculum in improving confidence, preparedness, and awareness

**Figure 6:**
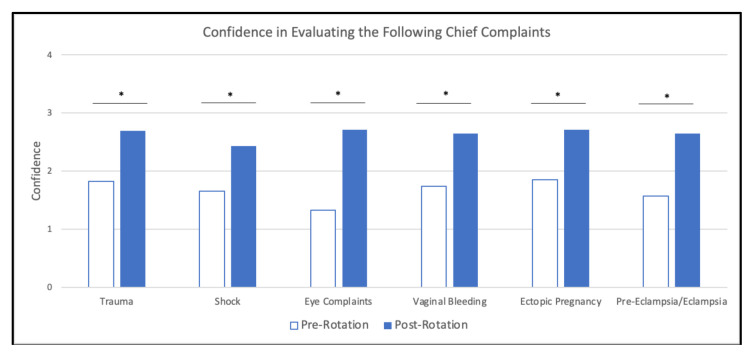
Confidence in Evaluating Chief Complaints

**Figure 7:**
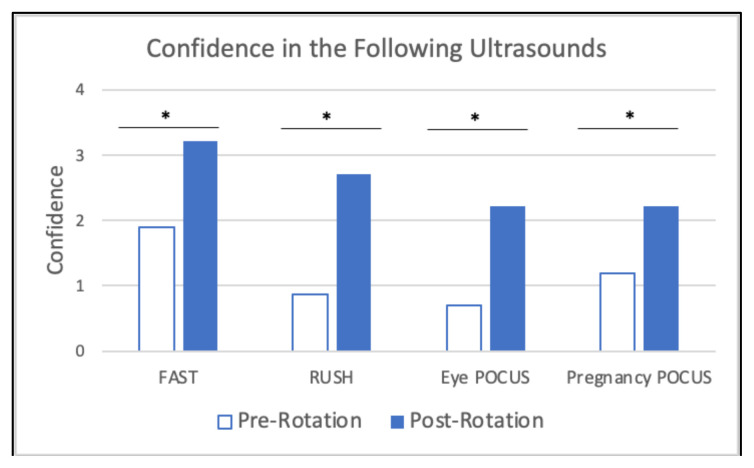
Confidence in Understanding Ultrasound

**Figure 8:**
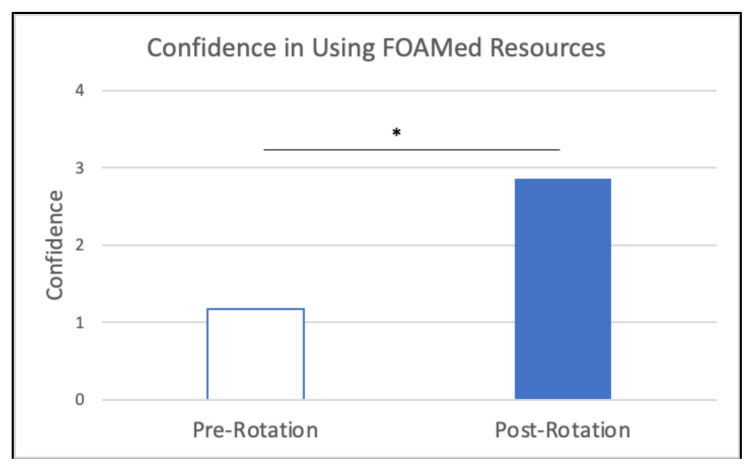
Confidence in Using FOAMed Resources

**Figure 9:**
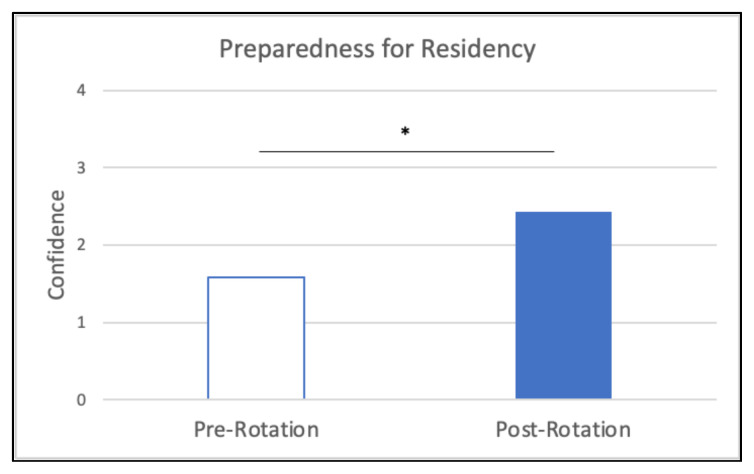
Self-Reported Preparedness for Residency

**Figure 10:**
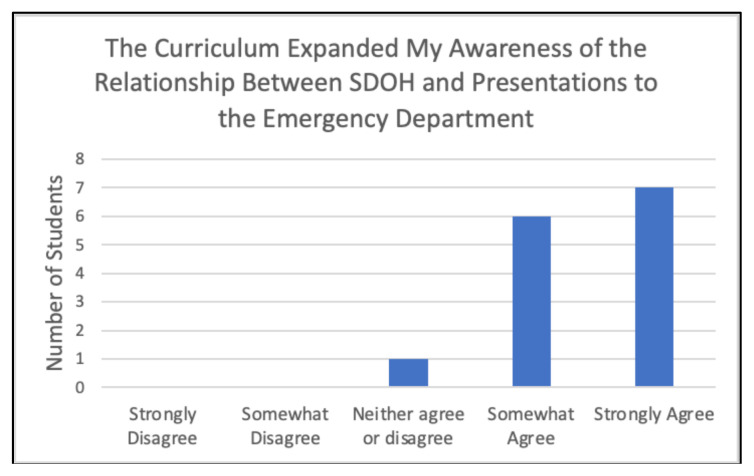
Awareness of the Impact of SDOH in the ED

When comparing pre-rotation and post-rotation responses, participants gained significant confidence in evaluating for the specified chief complaints (Figure 6), understanding specified point of care ultrasounds (Figure 7), and using emergency medicine resources (Figure 8) (*p<0.01). Participants also reported feeling significantly more prepared for residency at the end of the rotation compared to the start of the rotation (Figure 9) (*p<0.01). They also largely agreed that the curriculum expanded their awareness of the relationship between social determinants of health (SDOH) and presentations to the emergency department (ED) (Figure 10).

#### Learner satisfaction and general feedback

**Figure 11:**
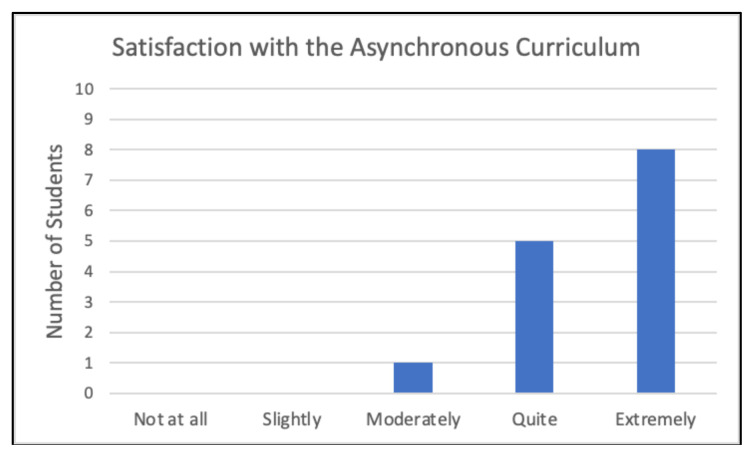
Satisfaction with the Asynchronous Curriculum

**Figure 12:**
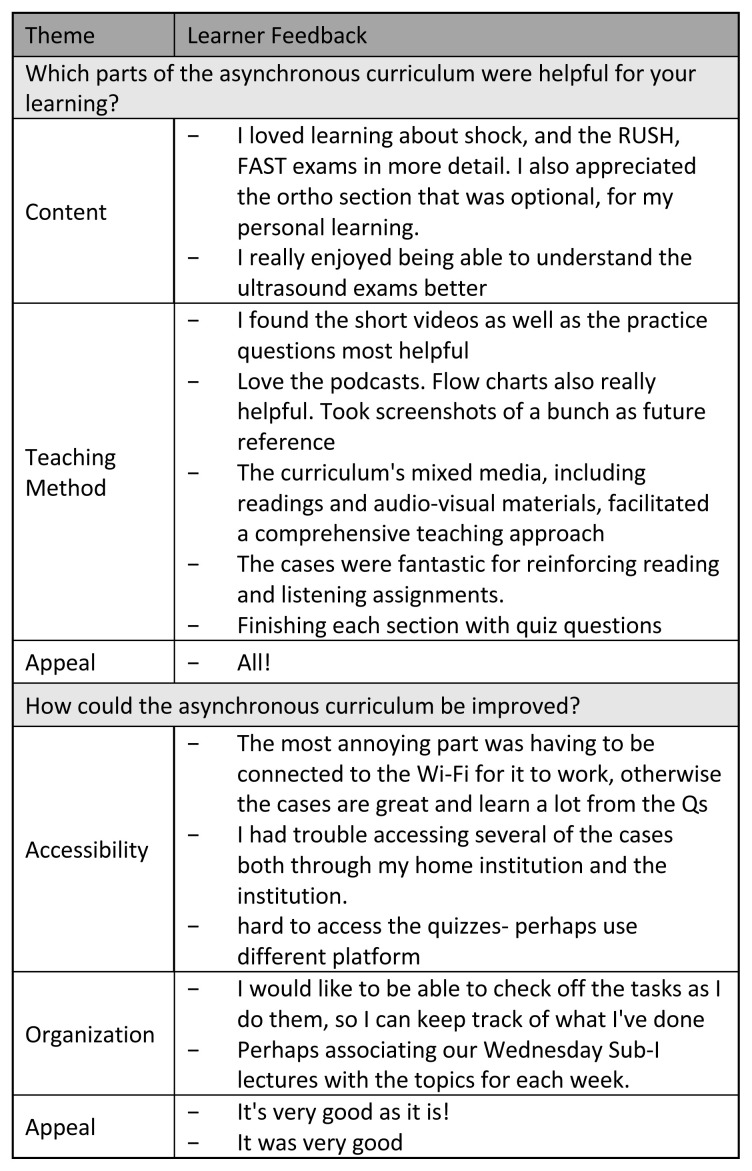
Learner Feedback

All participants expressed varying degrees of satisfaction with the asynchronous curriculum, the majority (57.1%) of which were “extremely” satisfied (Figure 11). No student responded that they were “slightly” or “not at all” satisfied. Regarding open-ended feedback, when asked about aspects of the curriculum that they found most helpful, students primarily commented on content and teaching methods used in the curriculum (Figure 12). More specifically, students reported enjoying the ultrasound content and the curriculum’s mixed media format. When asked about ways in which the curriculum could be improved, students commented on some difficulty accessing cases and practice questions and suggested ideas for organizing the curriculum (Figure 12). In both open-ended feedback sections, students commented positively on the appeal of the curriculum (Figure 12).

### Evaluation and Feedback

Early feedback that we received was that one of the resources was difficult to access. We received this feedback both through the curriculum’s surveys and through direct emails. This resource was not a free resource to the public, but because of our institution’s subscription to the resource, students were able to access it for free if they were on campus internet servers. This proved to be a problem for students who wanted to access the resource remotely and for students on an away rotation who had to navigate access to new servers. Because of this significant barrier, we replaced all of these resources with others that are similar in quality but more easily accessible. The original resources remain in the curriculum in the Addendum because this was the curriculum on which survey responses were based. It is important that institutions recognize this potential barrier, choose resources thoughtfully, and are adaptable as conflicts arise.

Another change that was made had to do with an unforeseen issue of using FOAM in our curriculum. The issue was that some of the links that were used to access certain resources changed or the resource itself became no longer available. In these cases, faulty links were replaced with working links or resources were replaced. Again, the original resources remain in the curriculum in the Addendum since this was the curriculum on which survey responses were based. Because of the constantly evolving nature of FOAM, we recognized the importance of resource monitoring to make sure the information provided to students is up to date and accessible.

Lastly, because a student commented on the desire to keep track of the completed items on the curriculum, we created a checklist and modified the layout of the curriculum on the website to assist with student organization. Institutions should also be cognizant of time demands on learners and choose a curriculum length that accommodates learners’ many responsibilities. While roughly two hours per module was suitable for our learners, length may need to be modified for other institutions.

## Figures and Tables

**Table:** 

Topic	Recommended Educational Strategy	Educational Content	Objectives	Learners	Timing, Resources Needed	Recommended Assessment, Milestones Addressed
Trauma	- Article and podcast describing the primary and secondary survey - Video about the eFAST exam - Podcast on the common interventions in a trauma resuscitation - Article on gun violence in America - Cases on trauma - Trauma practice questions	- How to prepare for incoming trauma patients - Detailed components of the primary and secondary survey - How to identify and manage common findings in trauma resuscitations - Proper technique and what to look for during an eFAST exam	The learner will be able to (1) describe the primary and secondary survey, and (2) identify common abnormal findings requiring timely intervention.	Sub-interns	2 hours Instructors: 0 Equipment: Device with internet access	Assessment: Self-assessment with cases and practice questions Curriculum assessment with surveys using Likert Scale Milestone: PC1
Eye complaints	- Podcast on evaluating eye complaints - Flowchart and notes summarizing the approach to vision loss - Videos on the slit lamp exam and ocular ultrasound - Article on the public health approach to managing diabetes - Cases on various eye complaints - Eye complaint practice questions	- How to gather an eye complaint history and perform an eye exam, including use of the ophthalmoscope, slit lamp, and ocular ultrasound - Explanation and differentiation of common and emergency eye pathology by history and exam	The learner will be able to (1) describe the major categories of eye complaints, common diagnoses, and can’t miss diagnoses, (2) gather an adequate history for eye complaints, (3) become familiar with the slit lamp exam, and (4) describe the functions of bedside ocular ultrasound.	Sub-interns	2 hours, 1 minute Instructors: 0 Equipment: Device with internet access	Assessment: Self-assessment with cases and practice questions Curriculum assessment with surveys using Likert Scale
Shock	- Video summarizing the pathophysiology of the four types of shock - Article, flowchart, and notes on undifferentiated shock - Video on the RUSH exam - Article on the racial bias of pulse oximeters - Cases on shock - Shock practice questions	- Explanation and differentiation of the four main types of shock - How to identify and manage the four main types of shock - Proper technique and what to look for during a RUSH exam	The learner will be able to (1) describe the types of shock and common causes of each, (2) determine appropriate workup for undifferentiated shock, (3) list the components of a RUSH exam, and (4) decide on management for each type of shock.	Sub-interns	2 hours, 7 minutes Instructors: 0 Equipment: Device with internet access	Assessment: Self-assessment with cases and practice questions Curriculum assessment with surveys using Likert Scale Milestone: PC1
OBGYN complaints	- Articles summarizing ectopic pregnancy and pre-eclampsia - Video and flowchart on the approach to vaginal bleeding - Article on point of care ultrasound in early pregnancy - Article on racial disparities in maternal health - Cases on OBGYN complaints - OBGYN practice questions	- How to form a differential for vaginal bleeding - How to identify and manage ectopic pregnancy, preeclampsia, and eclampsia - Proper technique and what to look for in a point of care ultrasound during early pregnancy	The learner will be able to (1) describe the clinical presentation of a ruptured ectopic, (2) develop an approach for evaluating vaginal bleeding, and (3) describe the clinical presentation and initial management of pre-eclampsia.	Sub-interns	2 hours, 26 minutes Instructors: 0 Equipment: Device with internet access	Assessment: Self-assessment with cases and practice questions Curriculum assessment with surveys using Likert Scale
Orthopedic complaints	- Article on the approach to orthopedic complaints - Article on the radiologic approach to the traumatic knee - Podcast on can’t miss knee complaints - Video on splinting techniques - Articles on when to call orthopedic consults - Cases on orthopedic complaints - Orthopedic practice questions	- Identification and management of patients with emergency orthopedic injuries - How to interpret knee x-rays - Basics on splinting common orthopedic injuries - Reasons to call orthopedic consults	The learner will be able to (1) develop an approach to common orthopedic complaints, (2) describe the indications, steps, and complications of splinting, (3) describe “do not miss” orthopedic diagnoses, and (4) determine when to call specialists.	Sub-interns	2 hours, 16 minutes Instructors: 0 Equipment: Device with internet access	Assessment: Self-assessment with cases and practice questions Curriculum assessment with surveys using Likert Scale
Ultrasound basics	- Videos on ultrasound basis - Article on ultrasound basics - Ultrasound basic practice questions	- Proper probe choice for your ultrasound exam - Physics of ultrasound - Identifying artifact vs pathology in ultrasound images - Using color and power doppler in your exam	*The learner will be able to (1) explain key principles of ultrasound physics and differentiate the functions of commonly used ED probes, (2) describe core clinical applications of bedside ultrasound in emergency medicine, (3) identify normal anatomy and interpret common pathologic findings on ultrasound images, (4) explore FOAMed ultrasound resources and integrate them into self-directed learning in emergency medicine	Sub-interns	1 hour Instructors: 0 Equipment: Device with internet access	Assessment: Self-assessment with practice questions Curriculum assessment with surveys using Likert Scale Milestone: PC12

* Learning objectives for ultrasound basics was unintentionally omitted from the original curriculum (for which data is provided) but was added to he curriculum’s subsequent iteration

## DIDACTICS AND HANDS-ON CURRICULUM

### Asynchronous Curriculum

#### Asynchronous Curriculum Introduction

This curriculum will begin to supplement content gaps in your didactic and clinical experience. The required weekly modules will cover and test basic medical knowledge of topics that are less often seen during clinical shifts at XXX. Optional modules are provided for further self-directed learning. Additional resources for core EM content can be found under the additional resources tab.

The modules also provide thought-provoking material regarding the social determinants of health and other factors that impact our patients’ health, their ED course, and overall trajectory. The juxtaposition of our two campuses provides a unique window to view the societal impacts on our patients’ health experience and outcomes. Our accompanying materials provide context and background to help you think about your patients beyond their acute ED chief complaint, and how their circumstances affect the care we provide.

##### Asynchronous Curriculum Objectives

- Prepare students to evaluate patients with presentations related to trauma, eye complaints, shock, and OBGYN
- Expose students to various EM resources that are available
- Expand students’ awareness of the relationship between social determinants of health and presentations to the emergency department
- Contribute to preparedness of sub-interns for residency

##### Schedule

Pre-Rotation Survey

Week 1: Trauma – 2h – Post-Module Trauma Survey

Week 2: Eye complaints – 2h 1m – Post-Module Eye Survey

Week 3: Shock – 2h 7m – Post-Module Shock Survey

Week 4: ObGyn – 2h 26m – Post-Module OBGYN Survey

Bonus Modules (optional): Ortho, Ultrasound Basics

Post-Rotation Survey

##### Each weekly module will include

- Learning Objectives
- Learning Resources: readings, podcasts, and videos covering basic concepts
- Cases: to apply the concepts learned, can be done individually or as a group
  ○ Go to Access Emergency Medicine: https://accessemergencymedicine.mhmedical.com/
  ○ Create a free account
  ○ Under “Cases” click on “Case Files in Emergency Medicine”
  ○ Throughout the modules, you will be asked to complete various cases from this list
  ○ Note that you will need to be on XXX network or VPN to access this resource
- Practice questions: to assess your understanding and identify areas for further self-directed learning
  ○ Go to Access Emergency Medicine: https://accessemergencymedicine.mhmedical.com/
  ○ Create a free account if you haven’t already
  ○ Under “Study Tools” click on “Review Questions”
  ○ You will have access to multiple question banks. Within these question banks, you will be able to create custom quizzes by topic
  ○ Throughout the modules, you will be asked to create quizzes by topic
  ○ Note that you will need to be on XXX network or VPN to access this resource
- Post-module survey: At the end of each module, we ask that you complete a post-module survey

#### Week 1 Module – Trauma

##### Objectives

- Describe the primary and secondary survey
- Identify common abnormal findings requiring timely intervention

##### Resources

- Choose one of the following resources to learn about the primary and secondary survey:
  ○ CDEM Reading -- Initial Trauma Assessment (15 min)
  ○ EM Basic Podcast -- Trauma Resuscitation Part 1 (25 min)
    ▪ Show Notes
- Watch this short video about the eFAST exam:
  ○ 5 Min Sono -- eFAST (5 min)
- Learn about common interventions in trauma resuscitation:
  ○ EM Basic Podcast -- Trauma Resuscitation Part 2 (25 min)
    ▪ Show Notes
- America’s unique gun violence problem, explained in 16 maps and charts (15 min)

##### Cases: work through this case

- Penetrating Trauma (17 min)

##### Practice Questions: take a custom 10-question quiz from the following section

- McGraw-Hill Specialty Board Review: Tintinalli's Emergency Medicine Examination & Board Review – 61 on trauma

**Post-Module Trauma Survey**

Total Time: 25m + 5m + 25m + 15m + 17m + 30m + 3m = 2h 0m

##### Bonus Cases (optional)

- Blunt Abdominal Trauma (20 min)
- Multisystem trauma and hypotension (15 min)

#### Week 2 Module – Eye

##### Objectives

- Describe major categories of eye complaints, common diagnoses, and can’t miss diagnoses
- Gather an adequate history for eye complaints
- Become familiar with the slit lamp exam
- Describe the functions of bedside ocular ultrasound

##### Resources

- Listen to this podcast (plus check out the show notes) for an overview of evaluating eye complaints:
  ○ EM Basic Podcast – Eye Complaints (40 min)
    ▪ Show notes
- Check out this flow chart and notes summarizing the approach to vision loss:
  ○ FoEM Flowchart – Approach to Vision Loss
  ○ FoEM Notes – Approach to Vision Loss
- Watch these short videos on the ED slit lamp exam and ocular ultrasound:
  ○ EM in 5 – Slit Lamp Anatomy (5 min)
  ○ EM in 5 – Ocular Ultrasound (5 min)
- Medical Care Alone Won’t Halt the Spread of Diabetes, Scientists Say (6 min)

##### Cases: work through the following cases

- Red Eye (20 min)
- Which is Which Set 01 (3 min)
- Which is Which Set 35 (3 min)
- Which is Which Set 38 (3 min)
- Which is Which Set 55 (3 min)

##### Question Bank: take a custom 10-question quiz from the following section

- Emergency Medicine PreTest Self-Assessment and Review - 19 on eye pain and visual change

**Post-Module Eye Survey**

Total Time: 40 + 5 + 5 + 6 + 20 + 12 + 30 + 3 = 2h 1m

#### Week 3 Module – Shock

##### Objectives

- Describe the types of shock and common causes of each
- Determine appropriate workup for undifferentiated shock
- List the components of a RUSH exam
- Decide on management for each type of shock

##### Resources

- Watch this short video summarizing the pathophysiology and treatment of the 4 types of shock:
  ○ EM in 5 Video -- The 4 Types of Shock (5 min)
- Read this article detailing an approach to undifferentiated shock:
  ○ EMCrit Reading -- Approach to Shock (15 min)
- Watch this video to learn the components of the RUSH exam:
  ○ 5 Min Sono Video -- RUSH (5 min)
  ○ *Note that more in depth videos about each of the components are available from the same page
- Check out this flowchart and note sheet summarizing the approach to undifferentiated shock:
  ○ FoEM Flowchart -- Approach to Undifferentiated Shock
  ○ FoEM Notes -- Approach to Undifferentiated Shock
- When it comes to dark skin, pulse oximeters fall short (4 min)

##### Cases: work through the following cases

- Shock Cases and Discussion Questions (30 min)
  ○ Shock Answer Key
- Hemorrhagic Shock (20 min)
- Sick, Shocked, and Sunburned (10-15 min)

##### Question Bank: take a custom 10-question quiz from the following section

- Emergency Medicine PreTest Self-Assessment and Review – 57 questions on trauma, shock, and resuscitation

**Post-Module Shock Survey**

Total time: 5 + 15 + 5 + 4 + 30 + 20 + 15 + 30 + 3 = 2h 7m

#### Week 4 Module – Obgyn

##### Objectives

- Describe the clinical presentation of a ruptured ectopic
- Develop an approach to evaluating vaginal bleeding
- Describe the clinical presentation and initial management of preeclampsia

##### Resources

- Read these summary articles on ectopic pregnancy and preeclampsia:
  ○ Core EM Reading – Ectopic Pregnancy (5 min)
  ○ Core EM Reading – Preeclampsia and Eclampsia (10 min)
- Watch this short video on an approach to vaginal bleeding:
  ○ Numose Vaginal Bleeding (8 min)
  ○ Check out this flow chart summarizing an approach to vaginal bleeding:
    ▪ FoEM Flowchart – Approach to Vaginal Bleeding
- Learn about POCUS in early pregnancy:
  ○ POCUS in early pregnancy (12 min)
- Why America’s Black Mothers and Babies are in a Life-or-Death Crisis (50 min)

##### Cases: work through the following cases

- Ectopic Pregnancy (18 min)
- 19 yo with vaginal bleeding (10 min)

##### Question Bank: take a custom 10-question quiz from the following section

- Emergency Medicine PreTest Self-Assessment and Review – 23 vaginal bleeding

**Post-Module OBGYN Survey**

Total time: 5 + 8 + 10 + 50 + 12 + 18 + 10 + 30 + 3 = 2h 26m

##### Bonus Case (optional)

- Ruptured Ectopic Pregnancy (25 min)

#### Bonus Module – Ortho (Optional)

##### Objectives

- Develop an approach to common orthopedic complaints
- Describe the indications, steps, and complications of splinting
- Describe “do not miss” orthopedic diagnoses
- Determine when to call specialists

##### Resources

- Read about the approach to orthopedic complaints:
  ○ CDEM Orthopedic Injuries (21 min)
- And now a deeper dive into a very common joint complaint:
  ○ *Radiologic Approach to the Knee (5 min)
- Learn what not to miss with another common complaint:
  ○ EM Basic Back Pain (27 min)
- Watch this video on basic splinting techniques:
  ○ NEJM Basic Splinting Techniques (13 min) - need to be on campus Wi-Fi or VPN
- And finally, learn when to call for help:
  ○ When to call ortho – Part 1 (10 min)
  ○ When to call ortho – Part 2 (15 min)

##### Cases

- 40 yo with low back pain (15 min)
- Northwestern Ortho Teaching Cases - 3, 7, and 8 (10 min)

##### Question Bank: take a custom 10-question quiz from the following section

- Emergency Medicine PreTest Self-Assessment and Review -- 33 on MSK injuries

Total Time: 21 + 5 + 27 + 13 + 10 + 15 + 15 +10 + 30 = 2h 16m

##### Additional Cases

- https://www.ortho-teaching.feinberg.northwestern.edu/cases/index.html

#### Bonus Module – Ultrasound Basics (Optional)

##### Resources

- 5 min sono: https://www.coreultrasound.com/5minsono-basics/
- CDEM Introduction to Bedside Ultrasound: https://www.saem.org/about-saem/academies-interest-groups-affiliates2/cdem/for-students/online-education/m3-curriculum/bedsideultrasonagraphy/introduction-to-bedside-ultrasound

##### Question Bank

- Emergency Medicine PreTest Self-Assessment and Review -- 28 on US in EM

#### Additional Resources

##### Navigating FOAMed

The information overload of EM resources can be overwhelming. Here is a guide by ALiEM on where to start: https://www.aliem.com/starters-roadmap-to-em-resources-books-websites-apps/

##### Core EM resources

Core EM content can be found at any of the following. For most, it works best to PICK ONE (or two) of these that you like and stick with it to cover the core key EM topics

- https://www.emclerkship.com/
  ○ Quick 10-minute videos that cover basics and are good as an intro but are not necessarily thorough
- http://embasic.org/
  ○ Podcast (with show notes) laying out an approach to most common chief complaints; later episodes branch out to more specific topics
  ○ Go to podcast archives where you can download a master file of topics / show notes
- https://foundationsem.com/frameworks/
  ○ Flow charts and notes for “Approach to common EM topics”
  ○ The main site also has “Learning Pathways” for common EM topics with lots and lots (maybe too many?) of resources linked in each pathway
- https://www.saem.org/about-saem/academies-interest-groups-affiliates2/cdem/for-students/online-education/m4-curriculum
  ○ Approach to common topics
  ○ Additional readings organized by topic
- https://www.emfundamentals.com/
  ○ Topic Pages – compilation of online resources on:
    ▪ True Emergencies
    ▪ Common Complaints
    ▪ Essential Skills
- https://www.aliem.com/bridge-to-em/
  ○ Free 8-week curriculum intended to prepare medical students for intern year
- https://www.emrap.org/c3
  ○ Available with purchase EMRA membership
- https://www.blog.numose.com/emed
  ○ Table of contents is organized by organ system
- Searching for specific topics
  ○ WikEM – best way to use is to google “wikEM + ______(whatever you are looking for)”
  ○ ALiEM – best way to use is to google “ALiEM + ______(whatever you are looking for)”
  ○ LITFL – best way to use is to google “LITFL + ______(whatever you are looking for)”
  ○ Numose search -- https://numose.com/search

##### Cases

- Access Emergency Medicine: https://accessemergencymedicine.mhmedical.com/cases.aspx?gboscontainerid=154
  ○ Note that you will need to be on XXX network or VPN to access this resource
- LITFL Clinical Cases: https://litfl.com/clinical-cases/
- Northwestern Ortho Teaching Cases: https://www.ortho-teaching.feinberg.northwestern.edu/cases/index.html

##### Questions

- Access Emergency Medicine Question Banks: https://accessemergencymedicine.mhmedical.com/qa.aspx
  ○ Note that you will need to be on XXX network or VPN to access this resource

### Needs Analysis Survey

1. I am currently an:
  Choices:
  - EM-bound M4
  - Other specialty bound M4
  - EM intern
  - Other ____
2. What type of curriculum did your sub-Is provide? How useful did you find it?
  - Required asynchronous
  - Optional asynchronous
  - Scheduled lectures
  - Procedures
  - Point of care ultrasound
  - Sim cases
  - Quizzes/Q banks
  Choices:
  - Not useful at all
  - Somewhat useful
  - Moderately useful
  - Very useful
  - Extremely useful
  - Not provided
3. What range of topics were covered? How useful did you find this?
  - Comprehensive EM topics
  - High yield topics
  - Specific topics to fill clinical gaps
  Choices:
  - Not useful at all
  - Somewhat useful
  - Moderately useful
  - Very useful
  - Extremely useful
  - Not covered
4. For each of the following topics, please mark whether:
  Choices
  - You encountered this topic on your clinical shifts at XXX
  - Recommended resources to review this topic would be useful during a 4 week sub-I
  - Altered mental status
  - Seizure
  - Stroke
  - Sepsis
  - Trauma
  - Cardiac Arrest
  - Anaphylaxis
  - Obgyn - ectopic, torsion, bleeding
  - Male GU complaints
  - Eye complaints
  - MSK/Ortho complaints
  - Pediatric EM
  - GI bleed
  - Hypertensive emergency
  - Shock, types of shock
  - Electrolyte abnormality
  - Toxicology/toxidromes
  - DKA (Diabetic ketoacidosis)
  - Sickle cell crisis
  - Point of care ultrasound
  - Radiology
  - ECGs
  - Common EM procedures
  - Rapid sequence intubation
  - Ventilators
  - Procedural sedation
  - Navigating FOAMed
5. While on your sub-Is, which resources were you aware of? Which resources did you find useful?
  - EMRA Pocketbooks
  - The Chief Complaint Handbook
  - Tintinalli’s Textbook
  - Rosen’s Textbook
  - EM Basic Podcast + Show notes
  - EM Fundamentals
  - EM:RAP
  - EM:RAP C3 (Comprehensive Core Curriculum)
  - WikEM
  - Academic Life in EM (AliEM)
  - UpToDate
  - EMCrit
  - EMDocs
  - EM Clerkship Podcast
  - Life in the fast lane (LITFL)
  - nuMose
  - nuMose ED
  - Foundations EM
  - Rebel EM
  - SAEM/CDEM M4 Curriculum
  - Ddx Of (specify)
  Choices
  - Found useful
  - Did not find useful
  - Not familiar with
6. Any other resources you would recommend?
7. What would be the ideal 4 week sub-I curriculum for you? (Eg, Optional vs mandatory? Comprehensive vs high yield vs specific topics to fill in gaps in clinical experience? What level of interactivity?)
8. Was there any part of a sub-I curriculum that was not good or that you found not helpful?

### Pre-Rotation Survey

1. I am an:
  Choices:
  - EM-bound M4
  - Other specialty bound M4
  - Other ____
2. How many 4-week EM Sub-Internships have you already completed?
  Choices:
  - 0
  - 1
  - 2
  - Other ____
3. How confident do you feel evaluating the following chief complaints/disease entities?
  - Trauma
  - Shock
  - Eye complaint
  - Vaginal Bleeding
  - Ectopic Pregnancy
  - Pre-eclampsia/Eclampsia
  Choices:
  - Not at all confident
  - Slightly confident
  - Moderately confident
  - Quite confident
  - Extremely confident
4. How confident do you feel explaining how to perform the following skills/procedures?
  - FAST exam
  - RUSH exam
  - Eye POCUS
  - Pregnancy POCUS
  Choices:
  - Not at all confident
  - Slightly confident
  - Moderately confident
  - Quite confident
  - Extremely confident
5. How confident do you feel navigating EM resources and FOAMed (Free Open Access Medical Education) to find answers to your clinical questions?
  Choices:
  - Not at all confident
  - Slightly confident
  - Moderately confident
  - Quite confident
  - Extremely confident
6. How prepared do you feel for residency?
  Choices:
  - Not at all prepared
  - Slightly prepared
  - Moderately prepared
  - Quite prepared
  - Extremely prepared

### Post-Trauma Module Survey

1. After completing this module, I feel more prepared to:
  - Describe the primary and secondary survey
  - Identify common abnormal findings requiring timely intervention
  Choices:
  - Strongly disagree
  - Somewhat disagree
  - Neither agree nor disagree
  - Somewhat agree
  - Strongly agree

### Post-Eye Module Survey

1. After completing this module, I feel more prepared to:
  - Describe major categories of eye complaints, common diagnoses, and can’t miss diagnoses
  - Gather an adequate history for eye complaints
  - Describe the functions of bedside ocular ultrasound
  - Feel more familiar with the slit lamp exam
  Choices:
  - Strongly disagree
  - Somewhat disagree
  - Neither agree nor disagree
  - Somewhat agree
  - Strongly agree

### Post-Shock Module Survey

1. After completing this module, I feel more prepared to:
  - Describe the types of shock and common causes of each
  - Determine appropriate workup for undifferentiated shock
  - List the components of the RUSH exam
  - Decide on management for each type of shock
  Choices:
  - Strongly disagree
  - Somewhat disagree
  - Neither agree nor disagree
  - Somewhat agree
  - Strongly agree

### Post-OBGYN Module Survey

1. After completing this module, I feel more prepared to:
  - Describe the clinical presentation of a ruptured ectopic pregnancy
  - Evaluate vaginal bleeding
  - Describe the clinical presentation and initial management of preeclampsia
  Choices:
  - Strongly disagree
  - Somewhat disagree
  - Neither agree nor disagree
  - Somewhat agree
  - Strongly agree

### Post-Rotation Survey

1. How confident do you feel evaluating the following chief complaints/disease entities?
  - Trauma
  - Shock
  - Eye complaint
  - Vaginal Bleeding
  - Ectopic Pregnancy
  - Pre-eclampsia/Eclampsia
  Choices:
  - Not at all confident
  - Slightly confident
  - Moderately confident
  - Quite confident
  - Extremely confident
2. How confident do you feel explaining how to perform the following skills/procedures?
  - FAST exam
  - RUSH exam
  - Eye POCUS
  - Pregnancy POCUS
  Choices:
  - Not at all confident
  - Slightly confident
  - Moderately confident
  - Quite confident
  - Extremely confident
3. How confident do you feel navigating EM resources and FOAMed (Free Open Access Medical Education) to find answers to your clinical questions?
  Choices:
  - Not at all confident
  - Slightly confident
  - Moderately confident
  - Quite confident
  - Extremely confident
4. Completing this curriculum expanded my awareness of the relationship between social determinants of health and presentations to the Emergency Department.
  Choices:
  - Strongly disagree
  - Somewhat disagree
  - Neither agree nor disagree
  - Somewhat agree
  - Strongly agree
5. How satisfied were you with the asynchronous curriculum as a whole?
  Choices:
  - Not at all satisfied
  - Slightly satisfied
  - Moderately satisfied
  - Quite satisfied
  - Extremely satisfied
6. How likely are you to change your clinical practice based on the following modules:
  - Trauma
  - Eye Complaints
  - Shock
  - OBGYN complaints
  Choices:
  - Not at all likely
  - Slightly likely
  - Moderately likely
  - Quite likely
  - Extremely likely
7. How prepared do you feel for residency?
  Choices:
  - Not at all prepared
  - Slightly prepared
  - Moderately prepared
  - Quite prepared
  - Extremely prepared
8. Which parts of the asynchronous curriculum were helpful for your learning?
9. How could the asynchronous curriculum be improved?
